# A rare cause of atypical facial pain

**DOI:** 10.1016/j.clinme.2024.100254

**Published:** 2024-10-18

**Authors:** Suzannah Hall, Kevin Michell, David Howlett

**Affiliations:** East Sussex Healthcare NHS Trust, Eastbourne District General Hospital, Eastbourne, United Kingdom

## Abstract

This case illustrates a rare cause of facial pain due to glossopharyngeal neuralgia in a 66-year-old male patient. Imaging confirmed an aneurysm of the cervical internal carotid artery as the cause; the aneurysm itself, likely secondary to an elongated styloid process (Eagle's syndrome). The imaging findings and management options are discussed below.

## Case presentation

**Fig. 1:**
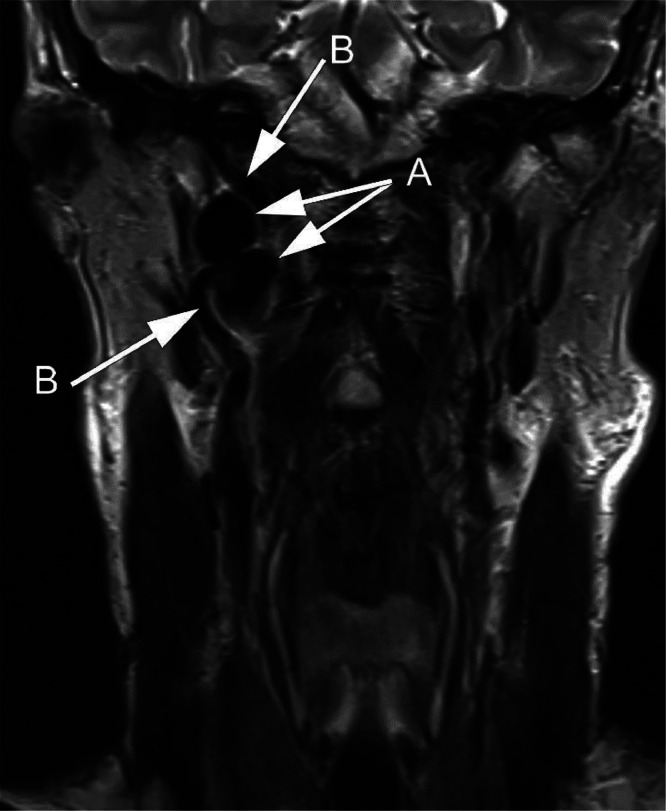
Coronal T2 W MR image through the neck demonstrates a bilobar mass, appearing of signal void (A), in the upper right neck near the skull base, the internal carotid artery can be seen entering and leaving the lesion (B).

**Fig. 2:**
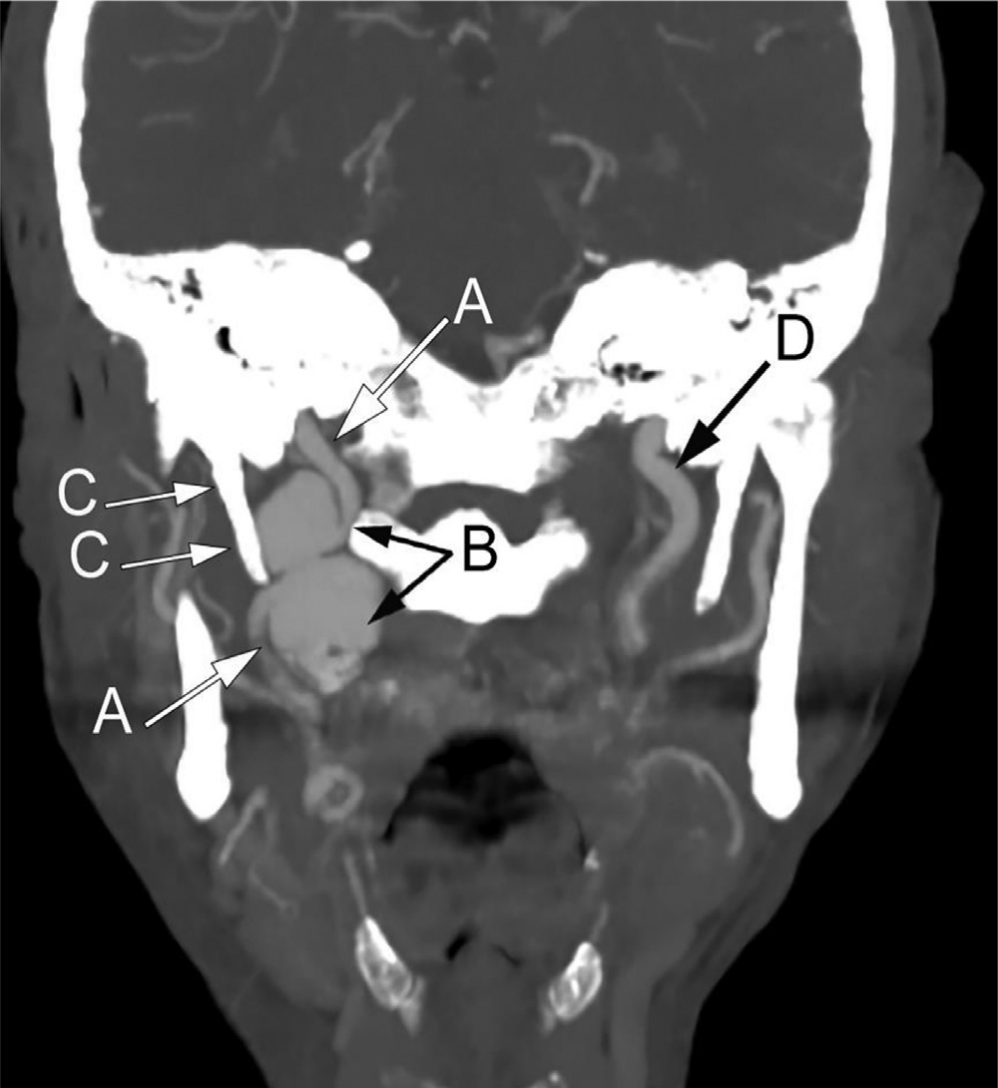
Coronal CT angiographic image demonstrates a normal calibre right internal carotid artery (A) proximal and distal to the aneurysm (B). Note elongated right styloid process (C) which impinges upon the aneurysm waist. Normal left internal carotid artery (D) with prominent left styloid process also present, although this appears remote from the vessel on this side.

**Fig. 3:**
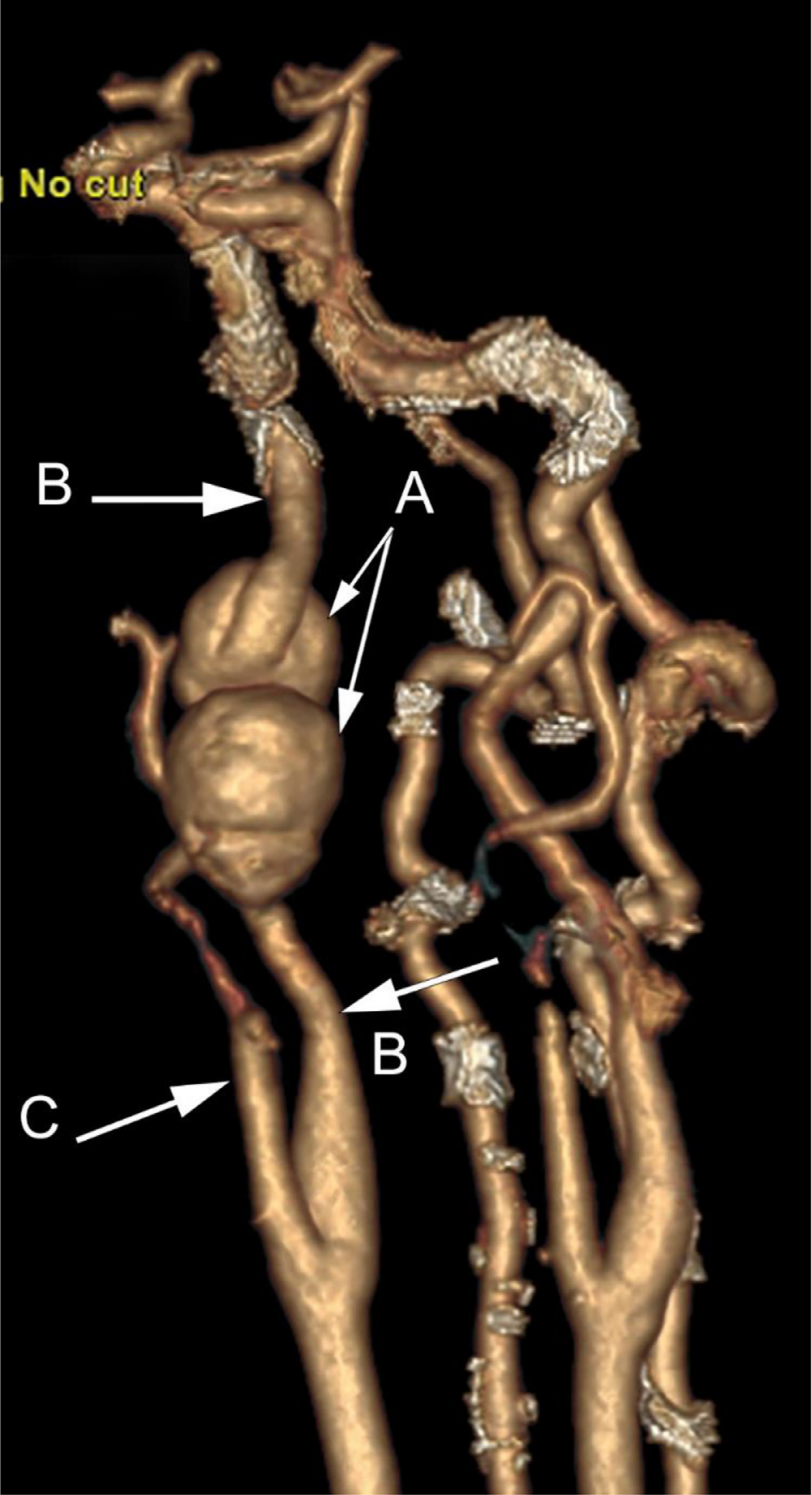
Multiplanar reformat (MPR) sagittal oblique image to show the cervical right carotid system confirms bilobar aneurysmal dilatation (A), normal calibre proximal and distal internal carotid artery (B), external carotid (C).

A 66-year-old male presented with a 5-month history of intermittent, sharp and stabbing pain in his right jaw and throat, radiating to the ear, worsened on mastication and swallowing. Clinical examination was unremarkable and a diagnosis of glossopharyngeal neuralgia was made. The patient proceeded to MR (magnetic resonance) imaging of the neck which demonstrated a large, bilobar aneurysm of the right internal carotid artery adjacent to the skull base (Fig. 1). Computed tomography angiography (CTA) confirmed the MR findings, but also delineated an elongated right styloid process impinging upon the narrowed waist of the aneurysm (Figs. 2, 3).

Glossopharyngeal neuralgia is a rare pain syndrome encompassing 0.2%–1.3% of cranial neuralgias.^1^ It most commonly arises secondary to brainstem neural compression in the posterior fossa, but also due to vascular causes, demyelination or tumour. In this case, neural compression occurring as the ninth nerve exits the skull base via the jugular foramen.^2^ Interestingly, ninth nerve compression in the neck can occur via a pathologically elongated styloid process (Eagle's syndrome), although in this case the elongated styloid process is likely the cause of the aneurysm itself.

Treatment options for extracranial carotid aneurysm include both surgical and endovascular options (the latter encompassing stenting and coil embolisation); in this case, conservative pain management was considered most appropriate in the first instance.

Treatment for idiopathic glossopharyngeal neuralgia, where no causative lesion is identified, includes both surgical and non-surgical management. Surgical procedures shown to be the most effective for glossopharyngeal neuralgia are rhizotomy and microvascular decompression.^3^ Surgical management needs careful consideration as although studies show these procedures drastically improve symptoms, known complications include permanent dysphagia and vocal cord paralysis.^3^

## CRediT authorship contribution statement

**Suzannah Hall:** Writing – original draft, Writing – review & editing. **Kevin Michell:** Writing – review & editing. **David Howlett:** Writing – review & editing.
